# Genetic Diversity, Pedigree Relationships, and A Haplotype-Based DNA Fingerprinting System of Red Bayberry Cultivars

**DOI:** 10.3389/fpls.2020.563452

**Published:** 2020-09-09

**Authors:** Bo Wu, Yun Zhong, Qianqian Wu, Fangyong Chen, Guangyan Zhong, Yiping Cui

**Affiliations:** ^1^Key Laboratory of South Subtropical Fruit Biology and Genetic Resource Utilization, Ministry of Agriculture, & Institute of Fruit Tree Research, Guangdong Academy of Agricultural Sciences (IFTR-GDAAS), Guangzhou, China; ^2^Citrus Research Institute of Zhejiang, Huangyan, China; ^3^Plant Protection Research Institute, Guangdong Academy of Agricultural Sciences, & Guangdong Provincial Key Laboratory of High Technology for Plant Protection, Guangzhou, China

**Keywords:** genetic diversity, single nucleotide polymorphisms, red bayberry, DNA fingerprinting, cultivar

## Abstract

High throughput sequencing was used to reveal the distribution of whole-genome variations in cultivated *Morella rubra* (Sieb. et Zucc.). A total of 3,151,123 SNPs, 371,757 small indels, and 15,904 SVs were detected in 52 accessions. Verification by Sanger sequencing demonstrated that the positive rate of the SNPs was approximately 97.3%. Search for more genetic variations was expanded to 141 red bayberry accessions, most of which were cultivars, by sequencing 19 selected genomic segments (SEG1-19). The results showed that each segment harbored, on average, 7.8 alleles (haplotypes), a haplotype diversity of 0.42, and a polymorphic information content (PIC) of 0.40. Seventy-two different genotypes were identified from the 141 accessions, and statistical analysis showed that the accessions with duplicated genotypes were either somatic mutants or simply synonyms. Core set selection results showed that a minimum of 34 genotypes could already have covered all the alleles on the segments. A DNA fingerprinting system was developed for red bayberry, which used the diversity information of only 8 DNA segments yet still achieved a very high efficiency without losing robustness. No large clade was robustly supported by hierarchical clustering, and well-supported small clusters mainly included close relatives. These results should lead to an improved understanding of the genetic diversity of red bayberry and be valuable for future molecular breeding and variety protection.

## Introduction

Red bayberry (*Morella rubra* Sieb. et Zucc.), also known as Chinese bayberry, is an economically important fruit crop that has been cultivated in China for more than two thousand years. Its fruit is not only very tasty but also rich in anthocyanins, vitamin C, and other antioxidant compounds (Ren et al., 2019). Red bayberry has a small genome size of ~323 Mbp, and three draft genomes of the species have been published recently (Jia et al., 2019; Ren et al., 2019). Red bayberry has separate male and female trees, which are diploid and propagate sexually under natural conditions. Its cultivars are all female and have been propagated asexually through grafting or cuttage in planting. Most red bayberry cultivars have been bred by crossing or selection of bud sports. In fruit, a bud sport usually denotes a branch phenotypically different from the rest of the plant which harbors one or more somatic mutations, and elite bud sport selection has played an important role in breeding new cultivars for many horticulture plants (Foster and Aranzana, 2018).

Hybridization and molecular breeding are believed to play more important roles in the improvement of red bayberry germplasm in the future. In this respect, much effort has been made to identify molecular markers associated with its important traits such as sex determination (Jia et al., 2019; Wang et al., 2020). Different types of molecular markers have been mined and applied, including amplified fragment length polymorphism (AFLP) (Zhang et al., 2009), inter-simple sequence repeat (ISSR) (He et al., 2010), simple sequence repeat (SSR) (Jiao et al., 2012; Jia et al., 2015; Wang et al., 2016; Wang et al., 2020), inter primer binding site (iPBS), and start codon targeted polymorphism (SCoT) (Chen and Liu, 2014). Liu et al. (2015) applied restriction site-associated DNA sequencing (RAD-seq) on pooled DNA of 18 individuals from 6 species (including *M. rubra*) of *Morella* and detected 8,360 single nucleotide polymorphisms (SNPs). However, there is still limited knowledge about the abundance of genetic variations in red bayberry at the whole-genome level.

Although a total of more than 300 red bayberry accessions have been recorded, including no less than 268 named cultivars (Liu et al., 2017), their genetic relationship has been a debating topic. First, the history of the domestication of red bayberry from wild populations is poorly understood. Wild populations of red bayberry have been found in subtropical forests not only in several provinces of China but also in Japan, South Korea, and the Philippines (Liu et al., 2017). Overlapping distributions between domesticated and wild red bayberry areas are quite common in China. Second, the genetic diversity and population structure of extant wild red bayberry have not been extensively studied. Research on the population structure of wild red bayberry in Guangxi province identified two main groups (He et al., 2010). More studies on the genetic diversity and putative clusters of red bayberry accessions yielded quite different results (Zhang et al., 2009; Jia et al., 2014; Jia et al., 2015; Wang et al., 2016). Moreover, the large clusters identified in these studies either had low (< 50%) bootstrap support or had no support rate given. Third, the origin of many cultivars has been undocumented, and it is difficult to distinguish cultivars selected by bud sports from those bred by crossing only based on the phenotypes. Some cultivars could have been simply renamed during transmission, which is difficult to confirm without the help of DNA fingerprinting.

Crop variety right protection has gained more and more attention in China, and a statistically reliable and efficient DNA fingerprinting system is in urgent need for red bayberry. SSRs have been the most used molecular marker type in DNA fingerprinting technology, for their convenience of usage and the high polymorphic information content (PIC) per marker (Gramazio et al., 2017). However, even though SNPs usually have lower polymorphic information content (PIC) per marker than SSRs, the usage of SNPs has been increasing dramatically in recent years due to several advantages (Fernandes et al., 2020; Hadizadeh et al., 2020; Wu and Alexander, 2020; Xin et al., 2020). SNPs are generally more abundant and more stable than SSRs, which are located at simple repeat regions and had mutation rates several orders of magnitude higher than SNPs (Fischer et al., 2017). Other advantages of SNPs include the easy application of automatic analysis and high consistency in genotyping results from different methods (Wu et al., 2014; Zhang et al., 2020). Moreover, methods that could genotype multiple SNPs simultaneously have been well developed (Tsykun et al., 2017), and the lower PIC per marker has become less a problem for SNPs. An easily applied and widely used method to genotype multiple SNPs simultaneously is by Sanger sequencing (Ezponda et al., 2019). Previously, we applied Sanger sequencing in genotyping 12 genomic segments of *Citrus maxima*, and the results showed that each segment contained multiple SNPs and harbored no less genetic diversity than SSRs (Wu et al., 2014). Taking advantage of the next-generation sequencing (NGS), genotyping by sequencing (GBS) could genotype hundreds to millions of SNPs simultaneously and has been widely applied in crop plants (Kim et al., 2016). In cucumber (*Cucumis sativus* L.), multiplex PCR amplification and GBS were applied for genotyping multiple genomic segments in the variety fingerprinting (Zhang et al., 2020). SNP-based fingerprinting systems have also been constructed recently in oolong tea (*Camellia sinensis* L.) (Lin et al., 2020), cacao (*Theobroma cacao* L.) (Mahabir et al., 2020), eggplants (*Solanum* L.) (Gramazio et al., 2017), and pineapple (*Ananas comosus* L.) (Zhou et al., 2015).

In this study, we report whole-genome genetic variations in red bayberry based on NGS of DNA mixture of 52 cultivars. The genotypes of 141 red bayberry accessions were revealed on 19 genomic segments. These data allowed us to perform genetic diversity, clustering, and pedigree relationship analysis. We also selected a core set of genotypes from the accessions to facilitate genetic diversity preservation and developed an efficient red bayberry DNA fingerprinting system.

## Materials and Methods

### Plant Materials and DNA Extraction

The leaves of 141 red bayberry accessions (**Supplemental Table 1**), including 140 female and one male accession (“Xiongzhu”), were collected from Field Genebank for Red Bayberry, Taizhou, Zhejiang Province, China. The leaves were ground into powder in liquid nitrogen, and whole-genome DNA was extracted using Quick Plant genomic DNA extraction kit N1193™ (Dongsheng Biotech, Guangzhou, China). DNA concentration and purity were assessed using NanoDrop™ 2000 Spectrophotometer (Thermo Scientific, Waltham, US). Obtained DNA solutions were required to have ≥50 ng/µl concentration, OD260/OD280 >1.6, and OD260/OD230 >1.8. The concentration of DNA solutions was adjusted to 50 ng/µl by adding distilled water. Agarose gel electrophoresis was carried out for the DNA extractions to make sure they had a low degradation rate.

### Next-Generation Sequencing

We added an equal amount (50 ng/µl × 5 µl) of DNA solutions from 52 of the 141 red bayberry accessions (**Supplemental Table 1**) into a 1.5 ml tube to get their mixture. A total of 100 µl DNA mixture and 200 µl of “Linhaizaodamei” DNA extraction were sent to BGI (Shenzhen, China) for next-generation sequencing. Pair-end sequencing libraries with an average of 500 bp insertion size were constructed for both the DNA mixture and “Linhaizaodamei”, which were subjected to sequencing on Illumina Hiseq 2000™ (San Diego, US). For scaffold assembling, another mate-pair sequencing library with an average insertion size of 10 kbp was constructed for “Linhaizaodamei”, and sequencing was carried out using the same approach. A total of 197.3 million clean read pairs (2 × 150 bp) were obtained for the DNA mixture, and 128.9 million (500 bp library) + 39.5 million (10 kbp library) clean read pairs (2 × 150 bp) were acquired for “Linhaizaodamei”. All NGS data have been submitted to NCBI under the bioproject ID PRJNA628691.

### De Novo Assembly of “Linhaizaodamei”

No red bayberry genome had been published at the beginning of our research. To acquire a draft genome for whole-genome variation discovery, we carried out the *de novo* assembly of the “Linhaizaodamei” genome with SOAPdenovo2 (Luo et al., 2012). Both sequencing reads of 500 bp and 10 kbp libraries were used in assembling contigs and scaffolds. We carried out assembling using kmer sizes from 55 to 85, and the rest parameters were set as default. The quality of assemblies using different kmer sizes was evaluated by QUAST 4.6.0 (Gurevich et al., 2013), and the assembly acquired with kmer size 79 had the largest contig and scaffold N50 sizes (**Supplemental Table 2**), which was used as a reference for selecting genotyping segments.

### Whole-Genome Variation Detection

Sequencing reads of red bayberry DNA mixture and “Linhaizaodamei” (500 bp library) were mapped to the female red bayberry reference genome (GCA_003952965.1) (Jia et al., 2019) using BWA v0.7.17 (Li, 2013). SAMtools v1.9 (Li et al., 2009) was used to transform the acquired SAM file into sorted BAM format. Small variants (including SNPs and < 50 bp indels) were called using the multi-allelic calling model by Bcftools v1.9 (Li, 2011). The sequencing depth and GC content in continuous 10 kbp windows across the reference genome were output by Bedtools v2.28.0. Several filters were applied in small variant calling: (1) only reads with mapping quality ≥25 and bases with base quality ≥20 were used; (2) SNPs and indels within 5 bp of another indel were discarded; (3) the non-reference allele had to be supported by at least 2 reads mapped to the forward strand and 2 reads mapped to the reverse strand of the reference; (4) genomic regions with <0.5 and >1.5 fold of average whole-genome sequencing depth were regarded as regions with abnormally low and high sequencing depth, and variants within these regions were discarded.

Whole-genome SVs were detected using Manta v1.6.0 (Chen et al., 2016). Five different types of SVs were called, including BNDs (translocation breakpoint), INVs (inversions), INSs (insertions of ≥ 50 bp novel sequence relative to the reference), DUPs (duplications), and DELs (≥ 50 bp deletions). The default filters of Manta were applied to filter low-quality SVs. To exclude false-positive SVs detected due to errors in the reference assembly, an additional requirement that the reference allele should be supported by at least four pairs of reads was also applied.

**Figure 1:**
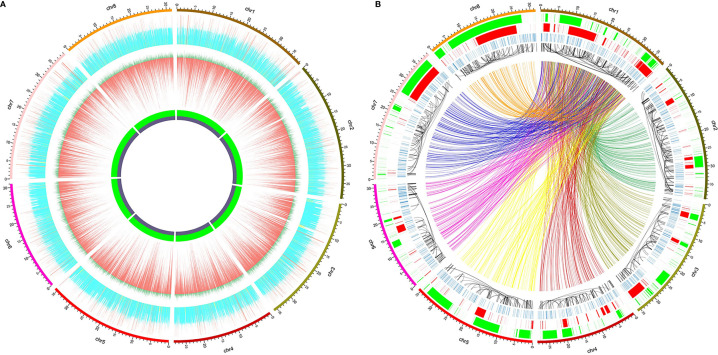
Distribution of whole-genome genetic variations identified from high-throughput sequencing of 52 red bayberry accessions. The 8 pseudo-chromosomes of the reference genome GCA_003952965.1 were shown as the outer ring in both **(A, B)**. **(A)** Distribution of normalized sequencing depth and the count of SNPs/indels across the eight pseudo-chromosomes. From inside to outside: the GC content panel, in which the heights of violet and green regions represent GC% and AT%, respectively; the SNP and indel distribution panel, in which the lengths of the red bars denote SNP counts and dark green bars denote indel counts; the normalized sequencing depth panel, yellow, blue, and red bars indicate windows with sequencing depth <0.5, between 0.5 and 1.5, and >1.5, respectively. **(B)** Distribution of SVs across the eight pseudo-chromosomes. From inside to outside: the BND panel (colorful links), only BNDs between Chr 1 and the other 7 pseudo-chromosomes were shown as links, which were assigned with different colors according to the pseudo-chromosome they bound to; the INV panel (black links), the black links pointed to the two ends of the inversion events; the INS panel (blue bands); the DUP panel (red bands); the DEL panel (green bands).

The circular graphs (**Figures 1A, B**) showing the distribution of small variants, SVs, and other information across the reference genome were drawn using Circos v0.69-8 (Krzywinski et al., 2009), and the data shown in the graphs were calculated in continuous non-overlapping 10 kbp windows across the eight pseudo-chromosomes. At most 400 SNPs and 60 indels were shown in **Figure 1A**, and greater counts were shown as 400 and 60, respectively. The sequencing depth of each window was normalized by the average whole-genome sequencing depth, and only 0 to 2 fold sequencing depths were depicted (> 2 fold were drawn as 2) in **Figure 1A**. All the variation data will be provided on request from the corresponding authors.

### Selection of Segments for Genotyping and Primer Design

Fifteen of the segments SEG1-19 (not including the four duplicated segments SEG7, 11, 14, and 15) were selected based on the “Linhaizaodamei” assembly. The DNA mixture sequencing reads were mapped to the assembly, and regions with >0.5 and <1.5 average whole-genome sequencing depth were filtered. The scaffolds of “Linhaizaodamei” assembly were ranged in descending order of scaffold size. To minimize the influence of linkage, the segments had better be physically distant from each other on the chromosomes, thus only one segment was designed on each of the largest 15 scaffolds. Primer3 was used to design the primers (Untergasser et al., 2012), and the melting temperatures (Tm) of the primers were set between 59°C and 61°C. The amplicon lengths were set between 700 and 1,200 bp, and the primer lengths between 18 and 25 bp. No indel was allowed in the amplified regions, and no SNP or indel was allowed in the primer regions. The rest four pairs of primers (SEG7, 11, 14, and 15) which amplified duplicated segments were designed based on GCA_003952965.1. Each of the segments had to have two copies in the reference, no indel was allowed, and the nucleotide similarity between the two copies of ≥99% when aligned with BLASTN. The primers for these four segments were designed using the same set of parameters used for the 15 segments by Primer3.

### Sanger Sequencing and Chromatograph Genotyping

PCR amplifications were carried out for the 141 accessions using primer pairs SEG1-19 (**Supplemental Table 5**) with the same conditions. Each reaction used 25 µl PCR solution with content suggested by TaKaRa Taq™ DNA polymerase (Takara Biomedical Technology, Beijing, China) manual. The amplification procedure included denaturation at 93°C for 3 min, 30 cycles of reaction (each cycle including 93°C denaturation for 30 s, 60°C annealing for 30 s, and then 72 °C extension for 1 m), and a final extension at 72 °C for 5 m. The specificity of the amplicon was checked by agarose gel electrophoresis, and only successful amplicons were subjected to Sanger sequencing by Tianyi Huiyuan (Beijing, China). The first primer of each primer pair listed in **Supplemental Table 5** was used in Sanger sequencing. When overlapped peaks, caused by indels, were observed in an accession, sequencing using the second primer would also be carried out.

The chromatograph files obtained from Sanger sequencing were subjected to genotyping using R package SangerseqR v1.22.0 (Hill et al., 2014). For non-duplicated and duplicated segments, the secondary to primary peak signal ratios were required to be higher than 50 and 30% to identify a putative heterozygous genotype, respectively. Heterozygous loci were output as IPUAC ambiguous bases in fasta format and subjected to multiple alignment using MUSCLE v3.8.1551 (Edgar, 2004). Then all variants and genotypes were output from the multiple sequence alignments using SNP-sites v2.5.1 (Page et al., 2016). The heterozygous loci were manually inspected in chromatographs, and false-positive variants located in poor quality regions were discarded.

### Haplotype Reconstruction, Genetic Diversity, and Pedigree Analysis

We reconstructed the haplotypes on SEG1-19 using the Bayesian inference (PHASE algorithm) implemented in DnaSP v6.12.03 (Rozas et al., 2017), with 10000 Markov chain Monte Carlo (MCMC) iterations run on each segment. A numeric ID was signed to each of the haplotypes on each segment. Based on the haplotype composition of each red bayberry accession, the diploid genotypes were represented by two haplotype IDs on each segment. From the cultivars with the same genotype, the best-known cultivar was chosen as the representative cultivar of the genotype. The cultivar in the brackets following a genotyping ID denoted its representative cultivar, and the number or alphabet in the brackets following a cultivar name denoted its genotype ID. The haplotype diversity, heterozygosity, and PIC were obtained by PowerMarker v3.25 (Liu and Muse, 2005) using the haplotype-based genotypes. The DSNs were calculated by comparing the genotypes (only one kept for each duplicated genotype) pairwisely.

The statistical power of SEG1-19 in parentage analysis under different conditions was calculated using Cervus v3.0.7 (Kalinowski et al., 2007) P1, P2, and P3. P1 is the non-exclusion probability for a false positive parent when the other parent is known, P2 is the non-exclusion probability for a false positive parent when no parent is known, and P3 is the non-exclusion probability of a false positive parent pair. Different genotypes that were not somatic mutants and shared at least one haplotype on each segment were identified as candidate parent-offspring pairs.

### Genotype Clustering and Core Set Selection

Genetic distance among the 72 genotypes was calculated using the simple matching method with 1,000 bootstraps and then subjected to hierarchical clustering (UPGMA) and NJ clustering (1,000 bootstraps) by DARwin v6.0.021 (http://darwin.cirad.fr/). A 1,000 bootstraps of UPGMA clustering was carried out in MEGA X (Kumar et al., 2018) using concatenated sequences of the genotypes on the SNPs. Principle component analysis (PCoA) was run on the genetic distance by DARwin v6.0.021.

For core set genotype selection, 2 to 34 different genotypes were selected by Core Hunter v3.2.1 (R package version) (Beukelaer et al., 2018) with allele coverage as evaluation measure. The method requires the selected genotypes to cover the largest possible number of alleles on all segments. The upper limit of the genotype number was set to 34 because the allele coverage had already reached 100%.

### The Statistical Power of Segments in Cultivar Identification

Using haplotype-based genotypes on SEG1-19, PI and PI_sib_ were calculated for the segments individually and cumulatively using GenAlEx v6.51b2 (Peakall and Smouse, 2012). The number of random individuals or siblings that could be reliably identified were computed using the same method applied in the study of Wu et al. (2014). Dropout v2.3.1.1 was applied to calculate the PI, PI_par/off,_ and PI_sib_ for each of the 72 genotypes.

## Results

### Whole-Genome Genetic Variations in Red Bayberry

High throughput whole-genome sequencing (NGS) was used to identify the whole-genome DNA variations in red bayberry. DNAs extracted from leaves of a total of 52 red bayberry (Yangmei) accessions were mixed and sequenced. A total of ~175 × sequencing reads were obtained and mapped to the female red bayberry reference genome (GCA_003952965.1). Genomic regions with abnormally high (> 1.5 fold average genome sequencing depth, 19,968,705 bp) and low (< 0.5 fold, 80,933,444 bp) sequencing depth were excluded from SNP and indel detection (**Figure 1A**).

A total of 3,151,123 SNPs and 371,757 small indels (shorter than 50 bp) were identified from the sequencing data. Among the variations, 2,883,745 SNPs and 339,513 indels were located on the 8 pseudo-chromsomes Chr 1–8, while the rest were located on 32,244 scaffolds which failed to be assigned to any chromosomes. The SNP density of the whole genome was thus averaged at 10.3 ± 9.2 SNPs/kbp. Comparatively, Chr 1 and Chr 6 had the highest (12.0 SNPs/kbp) and the lowest (9.1 SNPs/kbp) SNP densities, respectively. The transition/transversion ratio of all the SNPs was 2.04, and the most abundant SNP substitution types were C>T and G>A (**Supplemental Figure 1**).

Also, a total of 15,904 structural variations (SV) were identified from the sequencing data (**Figure 1B**), including ≥50 bp deletions (DEL) and ≥50 bp insertions (INS), duplications (DUP), recombination events or translocations (BND) and inversions (INV). Among the SV types, BND and DEL were the most abundant (**Supplemental Figure 2**). 6,111 BNDs, 638 INVs, 3,831 DELs, 1,433 INS, and 851 DUPs were located on the eight reference pseudo-chromosomes, while 2,049 BNDs, 52 INVs, 325 DELs, 121 INS, and 71 DUPs were located on the un-assigned scaffolds. The total length DELs and DUPs accounted for is 42.6% (119,275,719 bp) and 30.2% (84,348,026 bp) of the eight pseudo-chromosomes (**Figure 1B**).

### Genotyping 141 Red Bayberry Accessions on Multiple Genomic Segments

**Figure 2:**
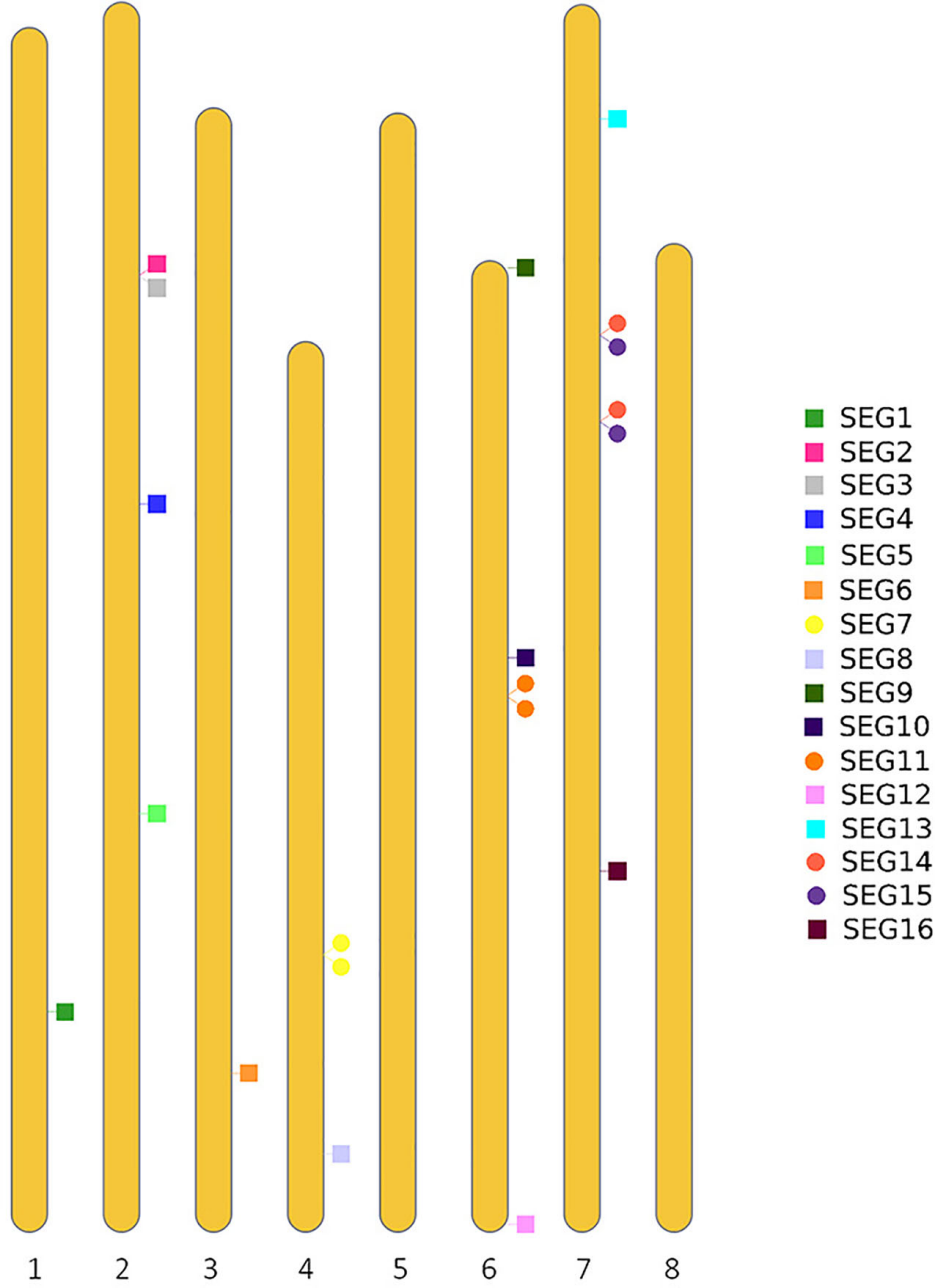
Distribution of the segments SEG1-19 on the pseudo-chromosomes of the reference genome.

To further reveal the genetic diversity of available red bayberry accessions, Sanger sequencing-based genotyping of 141 accessions was conducted using 19 pairs of primers (SEG1-19) (**Supplemental Table 1**). Four of the primer pairs (SEG7, 11, 14, and 15) were specifically designed to amplify adjacent duplicated (2-copy) genomic segments with nucleotide similarity > 99% in the reference genome (**Figure 2**). A total of 23 genomic segments with a total length of 13,506 bp were successfully amplified by the 19 primer pairs. Since the duplicated segments were immediately adjacent to each other on the chromosomes, they were therefore treated as one segment in genotyping. Hence the analyzed number of genomic segments was changed to 19. Sixteen (amplified by primer pairs of SEG1-16) genomic segments were located on six pseudo-chromosomes (**Figure 2**). Among the remaining three segments, SEG17 was aligned to a contig (RXIC01000053.1) that was not assigned to any pseudo-chromosome, but SEG18 and SEG19 were not homologous to any sequences in the reference genome. The SEG18 and SEG19 primer pairs were designed based on scaffold_893 and scaffold_6888 from our “Linhaizaodamei” genome assembly (**Supplemental Table 2**), respectively. Anyway, 8,818 and 5,327 bp of the 113,695 bp non-ambiguous bases (Ns) of the Scaffold_893 were aligned to Chr 6 and an unassigned contig (RXIC01000187.1) in the reference genome, respectively. Meanwhile, the scaffold_6888 could be partially (3,628 bp of the 7,031 bp non-ambiguous bases) aligned to the reference Chr 2 with high nucleotide similarity (> 99%).

**Figure 3:**
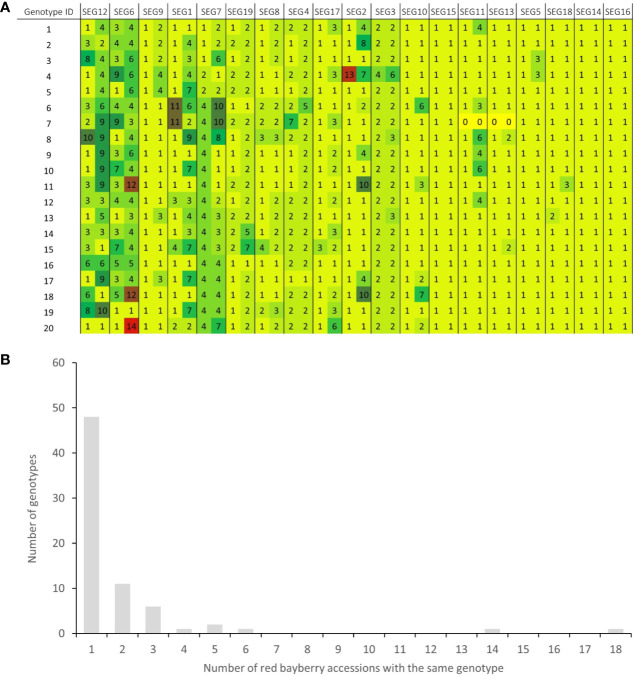
Haplotype-based fingerprints and genotype frequency in the 141 accessions. **(A)** Haplotype-based fingerprints of genotypes 1-20. The genotype IDs are the same as those listed in **Supplemental Table 1**, and the segments have been arranged in descending order of their PIC values. Different haplotypes on each segment are represented by different haplotype IDs and different background colors in the graph. **(B)** Distribution of genotypes in the 141 *M. rubra* accessions.

A total of 37 SNPs were identified on SEG1-19 from the NGS data of the 52 accessions, of which 36 were later verified to be authentic by Sanger sequencing, indicating that the *in silico* prediction of SNPs had an accuracy around 97.3%. A total of 176 high-quality SNPs were detected from the Sanger sequencing data of the 19 segments in the 141 accessions (**Supplemental Table 3**). The SNP density on the segments ranged from 6.2 SNPs/kbp (SEG11) to 20.9 SNPs/kbp (SEG8) and averaged at 13.8 ± 3.5 SNPs/kbp, with the minor allele frequency (MAF) ranging from 0.69 to 46.5%, and averaging at 8.6 ± 10.8%. Haplotypes were subsequently reconstructed through Bayesian inference on each segment among the 141 accessions. A total of 148 different alleles were identified, which had an average of 7.8 ± 3.5 haplotypes per segment (**Table 1**). The genotypes of all accessions were then assigned based on their haplotype composition. In the end, 72 different genotypes were identified among all 141 accessions (**Supplemental Table 1**, **Figure 3A**, and **Supplemental Figure 3**). The haplotype diversity of the 19 segments averaged at 0.40 ± 0.25 when calculated based on the 141 accessions and 0.42 ± 0.25 based on the 72 genotypes. Genotypes harbored various heterozygous loci, ranging from 0 to 14 heterozygous segments and 0 to 43 heterozygous SNPs (**Supplemental Table 1**). Notably, two different genotypes, 32 (“Longhaibaiyangmei”) and 16 (“Yongjiabaiyangmei”), were homozygous on all the segments. In contrast, 6 genotypes, E (“Yingsi”), 45 (“Yuelongsigukai”), 42 (“Nicimeibian”), 46 (“Xiangshanwuzi”), 48 (“Fugong No. 1”), and 3 (“Dahongpao”), had the highest amount of heterozygous SNPs.

**Table 1:** Mapping locations and polymorphic information of SEG1-19.

Segment ID	Corresponding genebank Accession No. and region	Pseudo-Chr ID	Amplicon Length (bp)	SNP number	HAP number	Hd_141^#^	Hd_72	PIC^^^
SEG1	CM012073.1:31443069-31443664	Chr 1	598	17	13	0.70	0.75	0.72
SEG2	CM012074.1:8725585-8726142	Chr 2	558	13	14	0.38	0.39	0.38
SEG3	CM012074.1:8745893-8746579	Chr 2	687	13	8	0.33	0.30	0.29
SEG5	CM012074.1:16028042-16027484	Chr 2	559	13	7	0.54	0.56	0.48
SEG5	CM012074.1:25918102-25918631	Chr 2	530	4	4	0.11	0.16	0.16
SEG6	CM012075.1:30844634-30845213	Chr 3	571	12	15	0.82	0.84	0.82
SEG7	CM012076.1:19596433-19597012; CM012076.1:19587959-19588538	Chr 4	580 * 2	15	10	0.79	0.78	0.75
SEG8	CM012076.1:25947801-25948231	Chr 4	431	10	8	0.50	0.53	0.50
SEG9	CM012078.1:219688-220407	Chr 6	720	14	9	0.41	0.41	0.37
SEG10	CM012078.1:12677973-12678627	Chr 6	654	6	7	0.24	0.29	0.29
SEG11	CM012078.1:13878476-13877755; CM012078.1:13927792-13927071	Chr 6	722 * 2	7	7	0.23	0.25	0.25
SEG12	CM012078.1:30785031-30785624	Chr 6	594	13	11	0.84	0.84	0.82
SEG13	CM012079.1:3648946-3648302	Chr 7	645	4	3	0.16	0.19	0.17
SEG14	CM012079.1:10551622-10552075; CM012079.1:13319714-13320167	Chr 7	454 * 2	1	2	0.09	0.11	0.12
SEG15	CM012079.1:10558954-10558414; CM012079.1:13327077-13326537	Chr 7	541 * 2	9	7	0.28	0.27	0.27
SEG16	CM012079.1:27686389-27686949	Chr 7	561	5	4	0.06	0.07	0.07
SEG17	RXIC01000053.1:100589-99925	NA^&^	665	10	7	0.49	0.50	0.45
SEG18	NA	Putative Chr 6	563	3	4	0.10	0.15	0.14
SEG19	NA	Putative Chr 2	576	7	8	0.59	0.61	0.54
Mean	NA	NA	594.1	9.3	7.8	0.40	0.42	0.40

^#^Hd_141 denotes haplotype diversity (Hd) calculated based on the 141 accessions, and Hd_72 is the haplotype diversity calculated based on the 72 genotypes; ^^^Polymorphic information content (PIC) was calculated based on the 72 genotypes; ^&^NA means not available.

### The Pedigree Relationship Among Red Bayberry Cultivars

**Figure 4:**
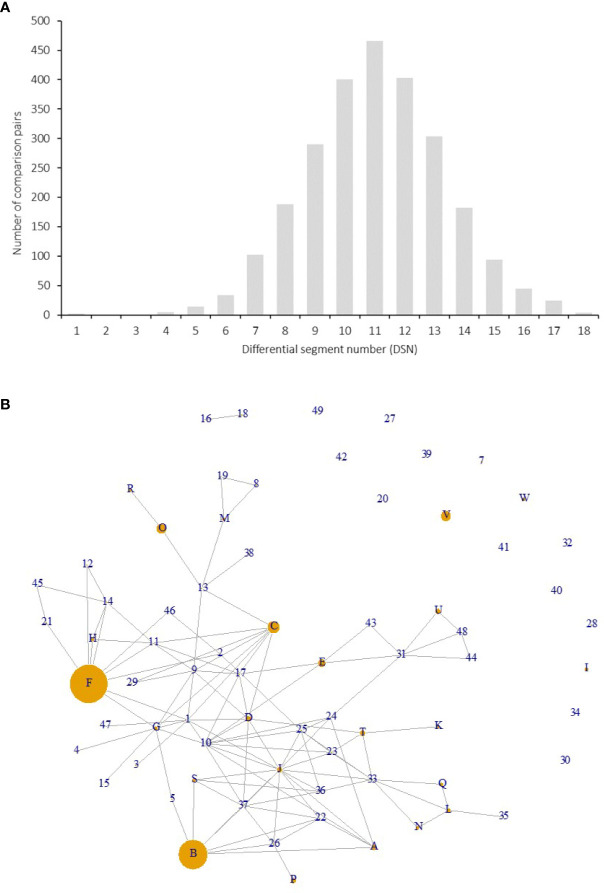
Differential segment numbers **(A)** and putative pedigree relationships among the 72 genotypes **(B). (A)** Distribution of DSNs among the 72 genotypes on SEG1-19**. (B)** Each node indicates a genotype, and each connection line indicates a putative parent-offspring relationship (direction unknown). The size of a node was proportional to the number of accessions with the same genotype in the 141 accessions.

Among the 141 accessions, known sport mutants generally shared the same genotype with their respective original cultivars on SEG1-19, and those with the same genotypes but with unknown origins were identified as putative synonymous cultivars. The 72 different genotypes were observed 1 to 18 times in the 141 accessions (**Figure 3B** and **Supplemental Table 1**). Forty-nine of them (genotype IDs 1–49) were detected only once, and 23 (genotype IDs A–W) occurred more than once. Surprisingly, 18 and 14 accessions shared the same genotypes with two elite cultivars, F (“Biqi”) and B (“Dongkui”), respectively. For instance, the genotype F was shared by the original variety “Biqi” and “Zaoqi” which was an early mature mutant of “Biqi”. The genotype B was shared by “Dongkui2”, “DK8”, “DK13”, “DK16”, “DK18”, “DB”, and “DB1”, and all of them originated as somatic mutants of “Dongkui”. An exception was observed between “Dingaomei” (C) and its somatic mutant “Dingaobian” (9). They shared the same genotypes at all segments except for the segment SEG9 on which “Dingaobian” was homologous whereas “Dingaomei” was heterozygous. It was therefore envisaged that a deletion had occurred to one allele of the SEG9 locus in “Dingaomei”, and it might be the event that gave rise to the new cultivar “Dingaobian”. Pair-wise differential segment numbers (DSN) among the 72 genotypes were no less than four (**Figure 4A**) except for that between “Dingaobian” (9) and “Dingaomei” (C). On average, the DSN among the different genotypes was 11.0 ± 2.3. Genotype 42 (“Nicimeibian”) had the largest average DSN (15.9 ± 1.1), while J (“Ruansi”) had the smallest average DSN (8.5 ± 2.5).

The putative parent-offspring relationship was identified through allele-sharing analysis on the SEG1-19 among the 72 genotypes (**Figure 4B**). The power of SEG1-19 in the identification of parentage was estimated by calculating their cumulative P1, P2, and P3, which denoted the statistical power of the set of markers in identifying parent-offspring relationships in different contexts (see Materials and Methods). The results showed that the cumulative P1, P2, and P3 were 9.0e-4, 2.9e-2, and 4.3e-6, respectively, showing that the 19 segments were statistically powerful in identifying parent-offspring pairs. All putative parent-offspring relationships among the genotypes were presented as links among the nodes (genotypes) in **Figure 4B**. The number of links possessed by different genotypes ranged from 0 to 14 and averaged at 3.2. The genotypes with the high number of links were J (“Linhaishuimei”, 14 links), 10 (“Unknown-1”, 11 links), I (“Xiangshandazhong”, 9 links), F (“Biqi”, 9 links), C (“Dingaomei”, 8 links), D (“Shizhutuzhong”, 8 links), G (“Linhaizaodamei”, 8 links), 33 (“Yewu”, 8 links), and 37 (“Zhenzhumei”, 8 links).

### Clustering of Red Bayberry Genotypes and Core Set Selection

**Figure 5:**
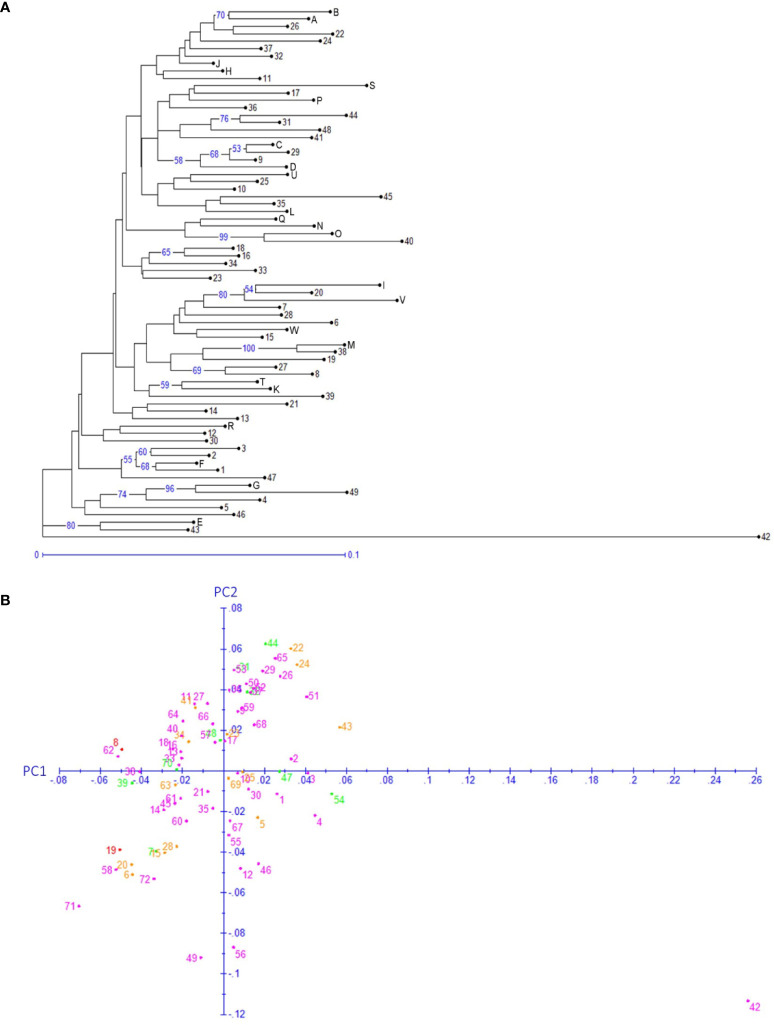
Genotype based neighbor-joining clustering **(A)** and principal component analysis **(B). (A)** The numbers on the branches denote the bootstrap support percentages, and only ≥50 support percentages have been shown. The lengths of the branches are proportional to the genetic distances. **(B)** Each dot indicates one of the 72 genotypes. Genotype IDs 1–49 are the same as those listed in **Supplemental Table 1**, and genotype IDs 50–72 corresponds to A–W in **Supplemental Table 1**. The color of the dots denotes the provinces of origin of the representative cultivars. Green, Fujian; orange, Hunan; purple, Zhejiang; red, Jiangsu.

Both neighbor-joining (NJ) and hierarchical clustering (UPGMA) methods were applied to the 72 genotypes based on the 176 SNPs in SEG1-19 (**Figure 5A** and **Supplemental Figure 4**). Genotype 42 (“Nicimeibian”) had the highest genetic distance from the other genotypes and was therefore used as the root. Neither NJ nor UPGMA robustly supported large clusters, but both of them well supported several small clusters containing only 2–3 genotypes. However, these clusters were not significantly more distant from the other genotypes, indicating low probability to been have derived from different ancestral populations. Most of these clusters contained interlinked genotypes, as shown by clades (31,44), (D, 9, 29), (A, B), (16, 18), (38, M), (K, T), (1, F), (4, G), and (43, E) in **Figure 4B**. The only male accession “Xiongzhu” (36) was not more divergent than the female cultivars to the rest of the accessions. Moreover, as shown in **Figures 5A, B**, no obvious correlation was found between the geographical origin (**Supplemental Table 1**) and genotype by using either the clustering or the principal component analysis (PCoA) method. In PCoA, the first two principal components (PC1 and PC2 in **Figure 5B**) combinationally accounted for 26% of the variance among the 72 genotypes.

**Figure 6:**
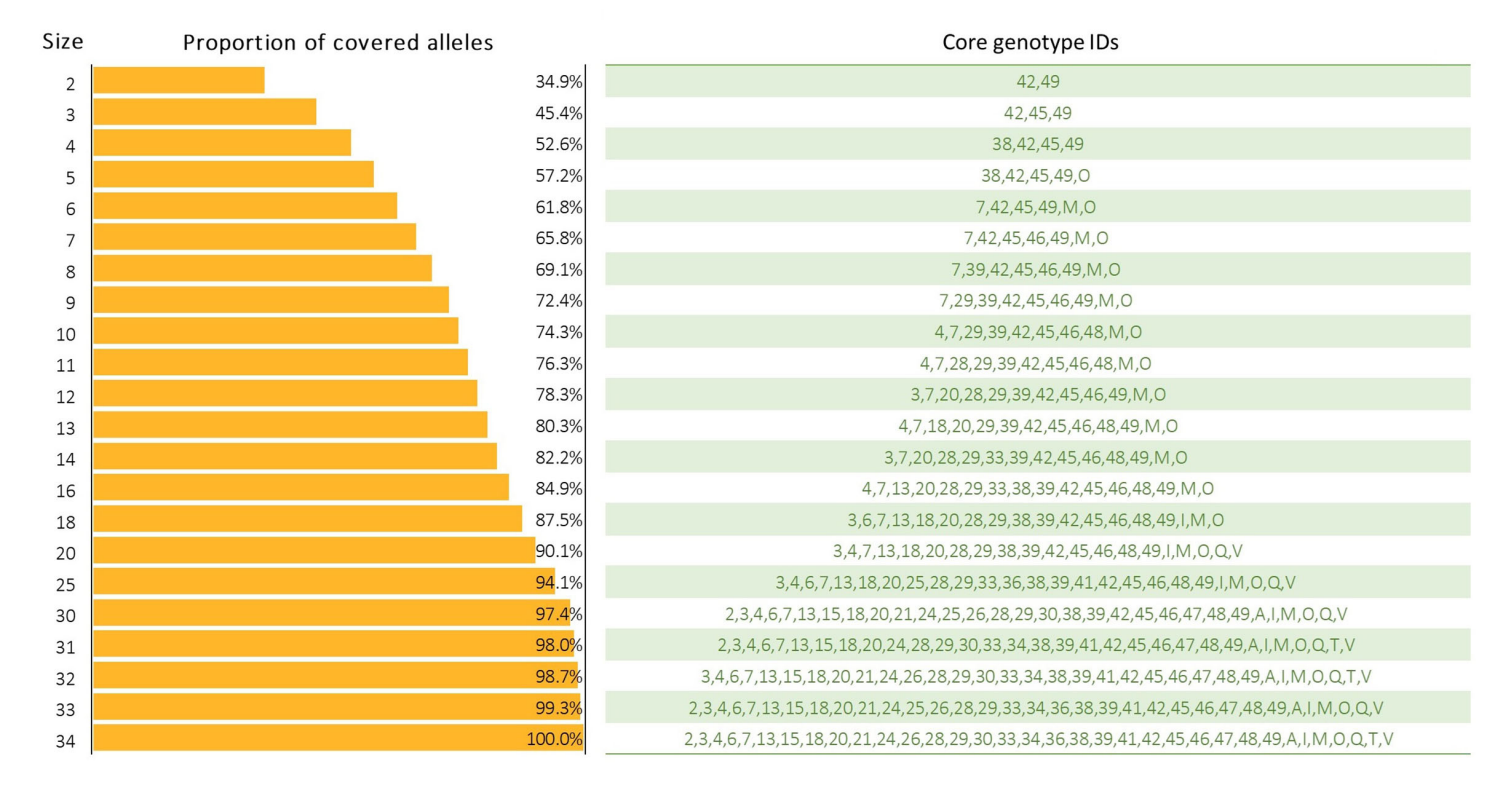
Core set selection. The number of genotypes required for core set selection and the subset of genotype IDs that cover most alleles.

A subset of genotypes harboring the highest proportion of alleles in the 72 genotypes was selected as a core set. The results showed that 13 genotypes were already enough to cover 80% of the total alleles on SEG1-19 (**Figure 6**). The coverage would increase to 90 and 100% if the genotypes chosen were increased to 20 and 34, respectively.

### Haplotype Based DNA Fingerprinting System

**Figure 7:**
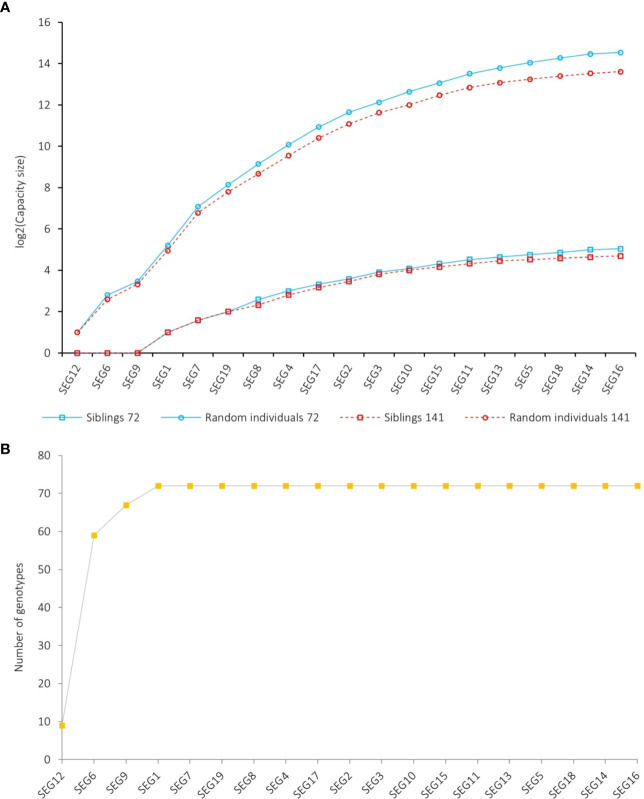
Discrimination capacity of SEG1-19 on red bayberry accessions. The segments shown in the graphs were arranged in descending order of their PICs. **(A)** Cumulative discrimination capacity of SEG1-19 on random individuals and siblings sharing both parents. Red and blue colors indicate the results based on the 141 accessions (duplicated genotypes included) and the 72 genotypes, respectively. **(B)** Cumulative number of genotypes observed on SEG1-19.

To investigate the power of using the diversity of SEG1-19 in identifying *M. rubra* cultivars, we started with an extreme situation that no clone was included in the 141 accessions. In that case, the PI probability of identity between two random individuals (PI) and the probability of identity between two siblings sharing both parents (PI_sib_) were 6.4e-10 and 1.5e-4, respectively (**Figure 7A**), indicating that the combined use of SEG1-19 could reliably distinguish 12,500 (2^13.6^) random individuals and 26 (2^4.7^) siblings. Therefore, it was possible to identify all the 14 members of the largest putative sibling group associated with the genotype J (**Figure 4B**) by using SEG1-19. In fact, there were very few if any, cultivars derived from siblings with the same parents in red bayberry. Taken together, it was concluded that those with the same genotypes were derived from cloning.

The discrimination capacity of the SEG1-19 could even be increased to 23,873 (2^14.5^) random individuals, and 33 (2^5.05^) siblings (**Figure 7A**), after removing the duplicated genotypes and taking the allelic frequency of the 72 genotypes into consideration. The PI, PI_par/off_ (probability of identity between a genotype and its parents or offspring), and PI_sib_ were calculated for each of the 72 genotypes. It was shown that SEG1-19 was very powerful in distinguishing an individual genotype from other randomly selected genotypes, and from its parents, offspring, or its siblings (**Supplemental Table 4**). It was clear that the discrimination power was quite different among the 19 segments since their PIC (polymorphic information content) varied from as low as 0.067 (SEG16) to as high as 0.82 (SEG12) (**Table 1**). After rearranging SEG1-19 in descending order of their PICs, it was clear that a minimum of 4 segments, the first 3 (SEG6, SEG9, SEG12) plus either SEG 7 or SEG1, were sufficient for identifying all 72 genotypes (**Figure 3A**, **Supplemental Figure 3**, and **Figure 7B**). Increasing the number of segments to 8 (**Figure 7A**) showed that the fingerprinting system could reliably distinguish 1,080 (2^10.1^) randomly sampled individuals, a number that greatly exceeded the total number of existing red bayberry cultivars.

## Discussion

Understanding the genetic diversity, especially the genome-wide genetic variations of red bayberry is of great importance in conventional and molecular-assisted breeding of red bayberry cultivars. By combined use of our high throughput sequencing data of 52 accessions and the reference draft genome of red bayberry (Jia et al., 2019), we were able to identify hundreds of folds more SNPs than those identified previously by using RAD-seq (Liu et al., 2015). To our knowledge, this was the first study on whole-genome variations in red bayberry. The results showed that the cultivated red bayberry accessions harbored a high density of SNPs (10.3 ± 9.2 SNPs/kbp). In contrast, SSRs that structurally belong to the category of indels were several orders of magnitude less than SNPs. The more abundant SNPs are more advantageous than SSRs in genetic map construction and association analysis in red bayberry. The density of the genetic variations was highly heterogeneous across the red bayberry genome (**Figure 1**), and the haplotype diversity varied significantly from 0.068 to 0.84 on SEG1-19, suggesting different genomic regions of red bayberry have been subjected to quite different selective pressures during evolution or domestication. Moreover, we also detected many SVs and large genome regions with abnormally low or high coverages. Accordingly, it could be inferred that some genome regions were highly divergent among red bayberry accessions and could not be represented by one reference genome.

Red bayberry has been domesticated by humans for more than 2000a, and thus it is difficult to infer the founder wild populations of the modern red bayberry cultivars. Previous clustering analysis of *M. rubra* accessions using data of AFLP (Zhang et al., 2009) and SSRs (Jia et al., 2014; Jia et al., 2015; Wang et al., 2016) yielded significantly inconsistent results, with clustered groups ranging from 2 to 6 groups. Although large groups were not well supported by all studies for supporting rates were either very low (< 50%) (Jia et al., 2014; Jia et al., 2015) or not provided (Zhang et al., 2009; Wang et al., 2016), several small groups were nevertheless well supported by SSR based clustering. In this study, small groups were also well supported and were clustered mainly according to their kinship or similar breeding history. Absence of support for large groups did not necessarily mean that the extant red bayberry cultivars have been univocally derived from one single wild population. It was also possible that enormous human breeding activity has well mixed the different original populations. Besides, no obvious correlation between genotypes of red bayberry cultivars and their geographical origins suggested that the germplasm had been extensively exchanged among different producing areas in China.

In this study, some red bayberry cultivars were identified to have the same genotypes based on SEG1-19 and were judged to be bud-sports or synonyms. This is no surprise since somatic mutants are widely observed in different fruit trees (Cabezas et al., 2011; Wu et al., 2014; Larsen et al., 2017) and selecting elite bud-sports has been an important breeding method in the history of fruit tree cultivar development (Foster and Aranzana, 2018). It is important to mention that synonyms could also harbor somatic mutations that have not altered phenotypes enough to define themselves as new cultivars. It has been known that synonyms and somatic mutants have rarely been distinguished from the original type with a limited number of markers (Nybom et al., 2014; Ruiz et al., 2019), as the chance for the mutation to be detected with a tested marker is low (Lee et al., 2016; Lamo et al., 2017). However, a deletion that caused the loss of heterozygosity on SEG16 distinguished “Dingaobian” (9) from its original type “Dingaomei” (C) in this study. In addition to known somatic mutants, a few undocumented synonyms were also identified in this study, suggesting that misidentification and undocumented renaming of cultivars did occur.

Our results also showed that different cultivars may have played different roles in the history. Some cultivars were more widely cultivated and some have played more important roles than others in breeding. The number of duplicated genotypes observed should be a good indicator of cultivar popularity. For instance, the two most widely grown cultivars Dongkui (B) and Biqi (F) had the largest number of accessions that shared their genotypes. Three other genotypes, C, O, and V, represented by three popular cultivars, Dingaomei, Chise, and Shangyubaiyangmei, were the second most observed in the 141 accessions. The links for putative parent-offspring relationship varied significantly among different genotypes (**Figure 4B**), suggesting these genotypes contributed differently to the current *M. rubra* cultivar pool in breeding. Among them, genotypes F (“Biqi”), C (“Dingaomei”), and B (“Dongkui”) not only possessed the most duplicated genotypes but also the largest numbers of putative parent-offspring links.

An efficient and statistically powerful DNA fingerprinting system is useful not only in breeding but also in breeder right protection. However, according to our knowledge, no DNA fingerprinting system has been constructed for red bayberry before this study. Previous genetic diversity analysis showed that AFLP (Zhang et al., 2009) and SSRs (Jia et al., 2014; Jia et al., 2015) could be highly polymorphic in red bayberry, but no fingerprinting system was established. The analysis in this study showed that segments containing multiple SNPs could have similar or even higher PICs than the SSRs reported in the previous studies (Jia et al., 2014; Jia et al., 2015). Our statistically powerful and highly efficient haplotype-based red bayberry DNA fingerprinting system should provide a better identification than the previously published marker systems in red bayberry.

## Data Availability Statement

The datasets presented in this study can be found in online repositories. The names of the repository/repositories and accession number(s) can be found in the article/**Supplementary Material**.

## Author Contributions

BW analyzed the data and wrote the manuscript. YZ, QW, and YC carried out the experiments. FC and GZ collected the plant materials. All authors contributed to the article and approved the submitted version.

## Funding

This work was supported by the 13th Five Year Plan of Chinese bayberry Breeding Special Project (2016C02052-2), the discipline construction of Zhejiang Academy of Agricultural Sciences (2018, No. 8), and the Taizhou Science and Technology Project (2020.03).

## Conflict of Interest

The authors declare that the research was conducted in the absence of any commercial or financial relationships that could be construed as a potential conflict of interest.
