# Supplementary figures and images for: Genetic Diversity, Pedigree Relationships, and A Haplotype-Based DNA Fingerprinting System of Red Bayberry Cultivars
Source: Front Plant Sci. 2020 Sep 9;11:563452. doi: 10.3389/fpls.2020.563452 (PMC7509436; doi:10.3389/fpls.2020.563452)

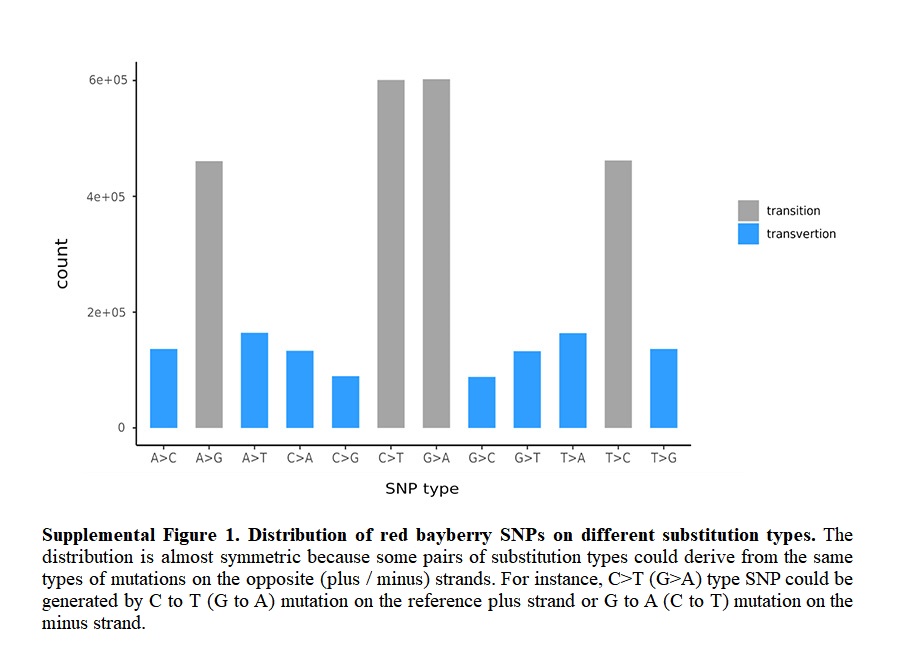

Supplement: Supplementary file 1 [file Image_1.jpg]

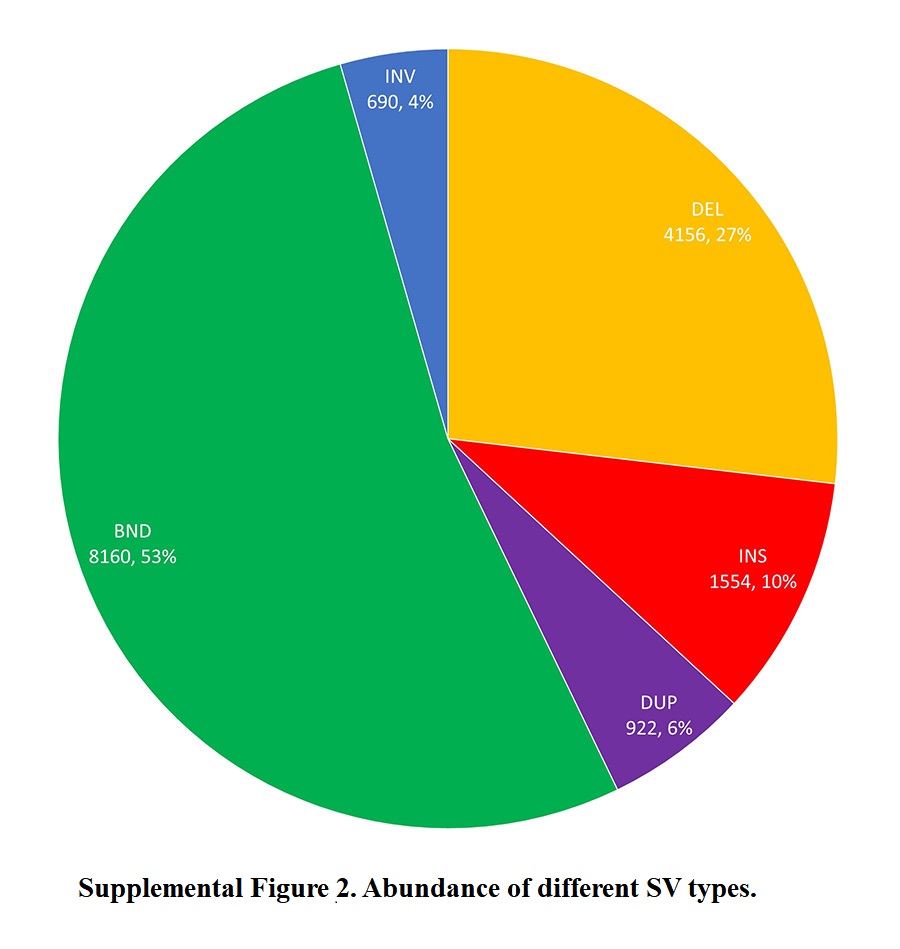

Supplement: Supplementary file 2 [file Image_2.jpg]

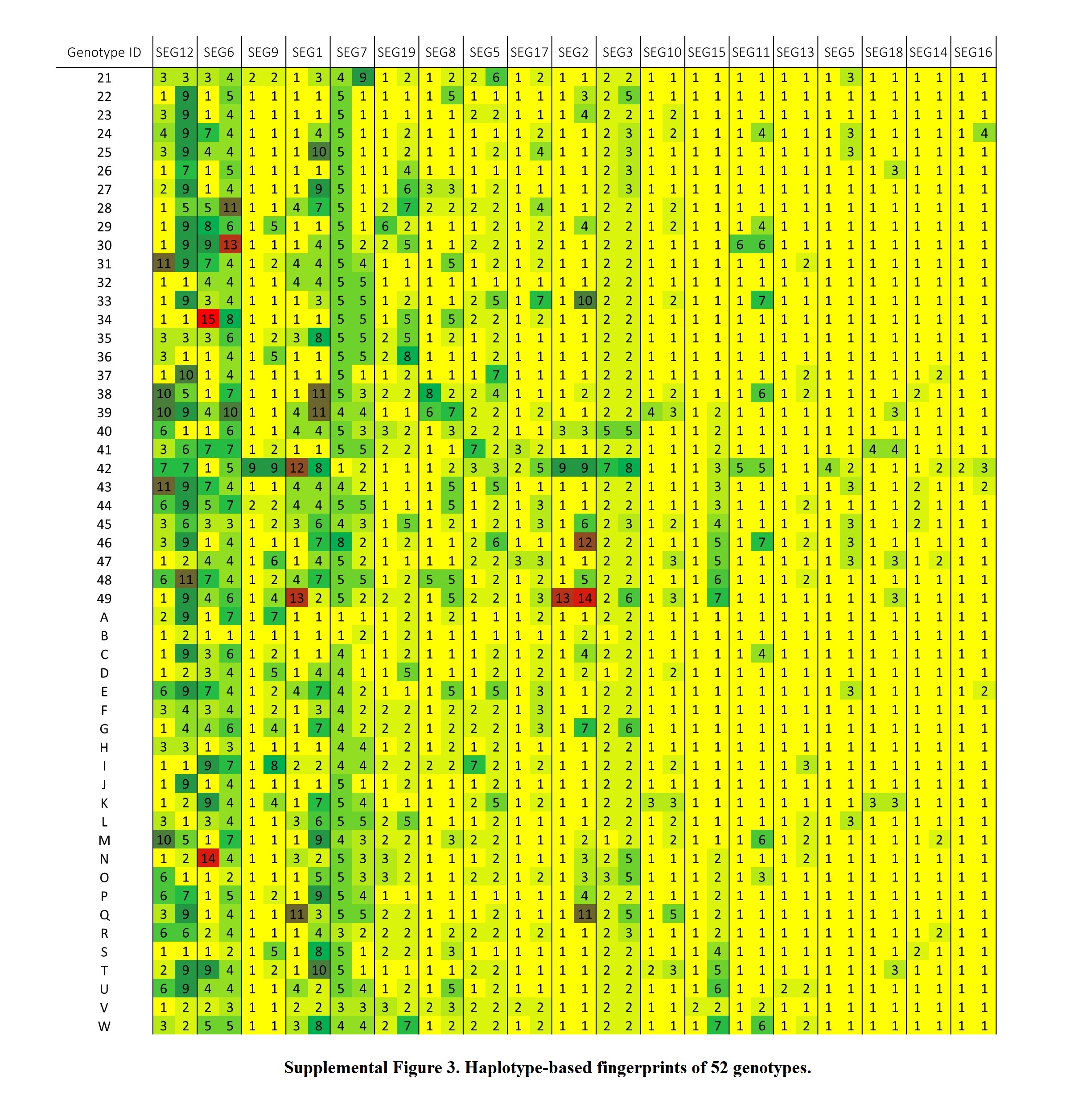

Supplement: Supplementary file 3 [file Image_3.jpg]

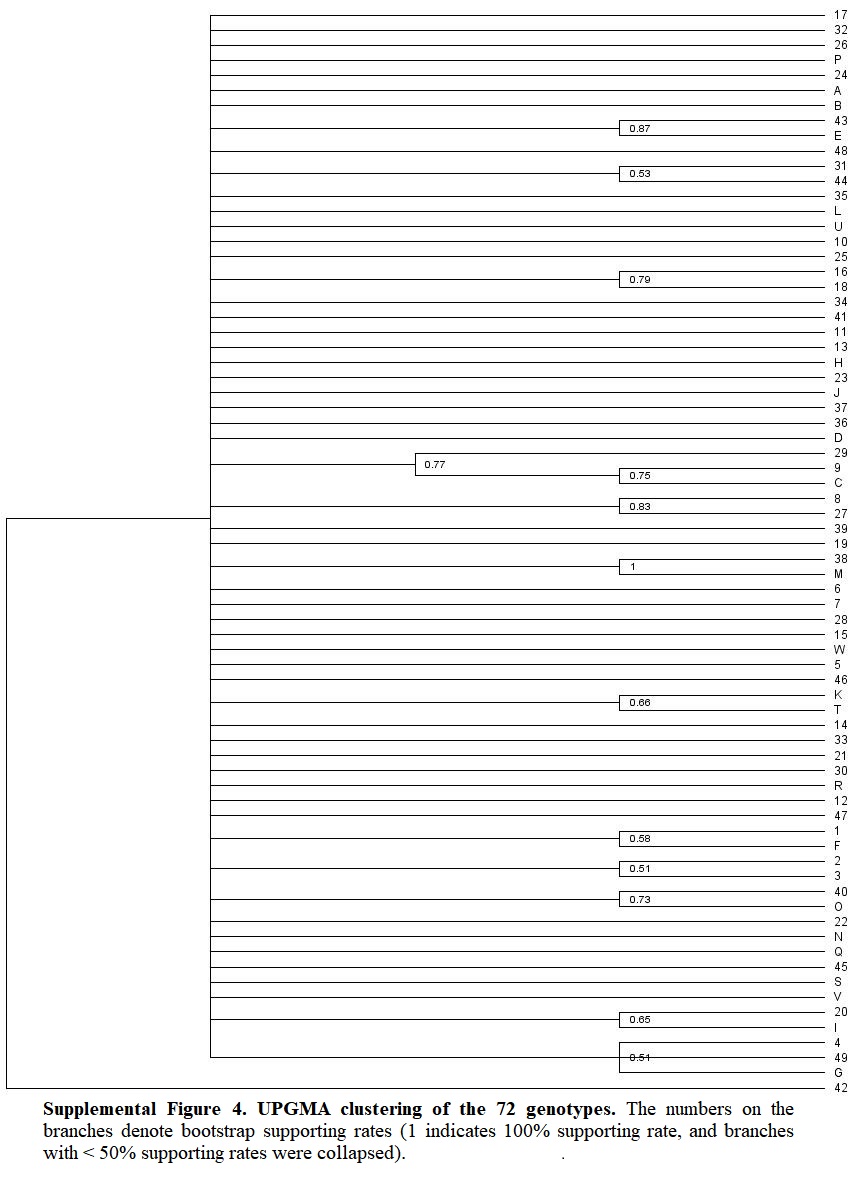

Supplement: Supplementary file 4 [file Image_4.jpg]
